# The reach and impact of social marketing and reproductive health communication campaigns in Zambia

**DOI:** 10.1186/1471-2458-7-352

**Published:** 2007-12-18

**Authors:** Ronan Van Rossem, Dominique Meekers

**Affiliations:** 1 Department of Sociology, Ghent University, Korte Meer 3, 9000 Gent, Belgium; 2 Department of International Health and Development, School of Public Health and Tropical Medicine, Tulane University, 1440 Canal Street, Suite 2200, New Orleans, LA 70112, USA

## Abstract

**Background:**

Like many sub-Saharan African countries, Zambia is dealing with major health issues, including HIV/AIDS, family planning, and reproductive health. To address reproductive health problems and the HIV/AIDS epidemic in Zambia, several social marketing and health communication programs focusing on reproductive and HIV/AIDS prevention programs are being implemented. This paper describes the reach of these programs and assesses their impact on condom use.

**Methods:**

This paper assesses the reach of selected radio and television programs about family planning and HIV/AIDS and of communications about the socially marketed *Maximum *condoms in Zambia, as well as their impact on condom use, using data from the 2001–2002 Zambia Demographic and Health Survey. To control for self-selection and endogeneity, we use a two-stage regression model to estimate the effect of program exposure on the behavioural outcomes.

**Results:**

Those who were exposed to radio and television programs about family planning and HIV/AIDS were more likely to have ever used a condom (OR = 1.16 for men and 1.06 for women). Men highly exposed to *Maximum *condoms social marketing communication were more likely than those with low exposure to the program to have ever used a condom (OR = 1.48), and to have used a condom at their last sexual intercourse (OR = 1.23).

**Conclusion:**

Findings suggest that the reproductive health and social marketing campaigns in Zambia reached a large portion of the population and had a significant impact on condom use. The results suggest that future reproductive health communication campaigns that invest in radio programming may be more effective than those investing in television programming, and that future campaigns should seek to increase their impact among women, perhaps by focusing on the specific constrains that prevent females from using condoms.

## Background

Zambia, like other Sub-Saharan African countries, is dealing with important health issues including family planning, reproductive health, and HIV/AIDS. Contraceptive use is low, reproductive health needs are not yet fully met, and AIDS is omnipresent [1-5]. In recent years, governmental and non-governmental organizations (NGOs) have designed and implemented social marketing and health communication programs to address these problems [5-9]. Although the focus of the programs varies – with some focusing more on family planning and others more on HIV/AIDS prevention – all of them could potentially increase condom use and in turn help reduce the number of new HIV infections. Several studies have examined the impact of specific programs [10,11], as yet the cumulative impact of a wider range of complementary programs has not yet been investigated. Responding to the growing importance of social marketing and health communication programs, the 2001–2002 Zambia Demographic and Health Survey (ZDHS) included a series of questions on exposure to such programs [2]. The purpose of this paper is to examine the reach of selected social marketing and reproductive health communication activities in Zambia, and to assess their cumulative impact on condom use.

### HIV/AIDS

Estimates of the adult prevalence of HIV in Zambia vary, with surveillance-based estimates putting the prevalence close to 20%, which is among the highest in sub-Saharan Africa [5,12,13]. HIV testing in the 2001–2002 ZDHS found a somewhat lower prevalence; 18% of women aged 15–49 and 13% of men aged 15–59 tested HIV positive [2]. HIV prevalence is more than twice as high in urban areas as in rural areas, 26% to 12%, respectively. Due to the relations between female commercial sex workers (CSWs) and long-distance truck drivers, HIV rates are extremely high along major national highways and border posts [14,15]. Recent estimates put HIV prevalence along major highways, borders, trading centers, and farming and mining towns in the vicinity of 30%. Among youth aged 15–24, the estimated HIV prevalence rate in 2001/2002 was 13% for men and 18% for women [16].

HIV/AIDS-related knowledge has improved considerably. Currently, approximately 95% of Zambians have heard of AIDS and know it is fatal. Nevertheless, a substantial fraction of Zambians continue to engage in risky sexual behavior. The 2001–2002 ZDHS indicates that 17% of men and 3% of women reported having two or more sexual partners in the past 12 months. Ten percent of men admitted paying for sex in the past 12 months [2].

Knowledge of male condoms is nearly universal, and more than three out of four adults know a condom source [2,5,17,18]. However, only 45% of females, in contrast with 72% of males, feel confident that they could obtain a condom. Despite the high HIV/AIDS awareness, levels of condom use have remained low, particularly with regular partners [1,2,19]. Only 8% of women and 10% of men reported using a condom in last intercourse with their spouse or cohabiting partner. By contrast, 33% of women and 44% of men reported using a condom in last intercourse with a non-cohabiting partner. Among men who admitted paying for sex in the past year, 45% reported using a condom at last paid sex [2]. Similarly, high-risk groups, such as truck drivers and CSWs, typically do not use condoms consistently [14].

### Social marketing and reproductive health communication campaigns

Since the mid-1980s, numerous approaches have been used to address these reproductive health problems and to prevent the spread of the HIV virus. At present, numerous reproductive and HIV/AIDS prevention programs are being implemented through the private, public, and voluntary sectors [3,20-24]. These programs target both the general population and high-risk groups such as women, adolescents, truck drivers, and commercial sex workers. Several programs specifically target adolescents and young adults [25-29].

Most programs use mass media and/or interpersonal communication campaigns to encourage Zambians to use contraceptives, and to prevent the spread of the HIV virus by promoting condom use and safer sexual behavior.

The Zambia Social Marketing Program (ZSMP) was launched in 1992, and is implemented by the Society for Family Health (SFH), an affiliate of Population Services International. SFH distributes subsidized *Maximum *brand condoms, and promotes their use through intensive mass media and interpersonal communications. Like several other organizations, SFH makes extensive use of radio and television to promote healthy behavior, including use of *Maximum *brand condoms. For example, SFH's *Club NTG *(New Teen Generation) is a youth-oriented radio program about issues that affect youth, such as teen pregnancy, HIV/AIDS, sexuality and condom use. Similarly, *An Inside Look *is an interactive television talk show that addresses health and social issues [9]. SFH also produced and broadcast radio and television public service announcements with Dr. Kenneth David Kaumba, the former President of Zambia. These public service announcements aim to discourage HIV-related stigma, and promote faithfulness, condom use, and voluntary testing and counseling [8].

Many Zambian health campaigns have made extensive use of print media, song, dance, and drama, but recently radio and television programs have become popular means of educating the public about family planning and HIV/AIDS prevention [5,6]. The Zambia Integrated Health Programme (ZIHP) produces several health-related radio and television programs. The ZIHP radio and television documentary series entitled "Your Health Matters" is broadcast on the Zambia National Broadcasting Company's (ZNBC) Radios One and Two and on television. The documentaries cover topics such as how HIV/AIDS is affecting Zambian youth, how youth can change their behavior, and how teachers and parents can help youth resist the pressure to have sex [30]. "Our Neighbourhood" is a 26-week 30-minute radio distance education program that focuses on health promotion and community mobilization. The program is produced by ZIPH and is broadcast four times each week, in Zambia's five major languages [31]. ZIHP also produces the television program X-plosion. X-plosion is a four-episode variety show focusing on HIV/AIDS. The show uses national and international musicians to promote HIV/AIDS awareness and prevention [32]. "Lifeline Zambia" was created by World Touch Ministries, and uses radio to highlight HIV/AIDS issues through testimonials from real people [33]. The popular South African drama series *Soul City *is also broadcast in Zambia. *Soul City *uses both television and radio soap opera series to educate the public about HIV/AIDS [7,34]. The *Soul City *TV drama comprises 13 episodes of 30 minutes each, while some of the radio series consist of series of either 45 or 60 episodes of 15 minutes each. The series addresses misinformation about HIV/AIDS and tackles complex topics such as the stigma associated with HIV infection.

## Methods

### Data

This study uses data from the 2001–2002 Zambia Demographic and Health Survey (ZDHS), which contains information on a nationally representative sample of 7,658 women aged 15–49 and 2,145 men aged 15–59. The survey was implemented by the Central Statistical Office and the Central Board of Health of Zambia. The survey instruments collected information on a wide range of topics, including mass media exposure, family planning, fertility, and HIV/AIDS/STI-related knowledge and behavior [2].

In addition to the standard question modules, the ZDHS includes questions on exposure to social marketing and health communication activities about family planning and HIV/AIDS. Specifically, both female and male respondents were asked if they had listened to any of the following four radio programs in the past six months: 1) *Your Health Matters*, 2) *Lifeline*, 3) *AIDS and the Family*, and 4) *Our Neighborhood*. They were also asked if they had watched any of the following four television programs in the past six months: 1) *Your Health Matters*, 2) *Lifeline*, 3) *Soul City*, and 4) *X-plosion*. In addition, male respondents were asked if they had ever seen or hear any messages about *Maximum *brand condoms, and if so, where they had seen or heard such messages.

The data were collected using a three-stage sampling design. First, 320 clusters were selected from the 2000 Population Census. Next, a representative sample of approximately 8,000 households was selected from those clusters. All women aged 15–49 in the selected households were eligible for interviewing. In a sub-sample of one-third of households, all men aged 15–59 were eligible for interviewing [2]. For the analyses, we used the non-weighted samples. Only sexually experienced respondents are included in the analyses.

### Measures

The outcome measures, capturing the respondents' reproductive health behaviors, for our analyses are dichotomous variables indicating whether the respondent had ever used a condom, and had used a condom during the last sexual encounter. These outcome variables were calculated only for the sexually active respondents.

Our indicators of exposure to communication programs about family planning and HIV/AIDS include a count of the number of radio programs that the respondent heard in the previous six months, ranging from 0 to 4 (including *Your Health Matters*, *Lifeline*, *AIDS and the family*, *Our Neighborhood*) and a count of the number of television programs she or he heard, ranging from 0 through 4 (including *Your Health Matters*, *Lifeline*, *Soul City*, *X-plosion*), as well as a count of the total number of programs they had seen or heard (range 0–8). For men, we also include an indicator of the number of sources of information about *Maximum *brand condoms.

As control variables, we included the respondents' age (in years), place of residence (Lusaka, other urban, or rural), religion (Protestant, Catholic, or Other), highest level of education achieved (none, primary, secondary or higher), number of sexual partners in the past 12 months, and perceived personal risk for HIV/AIDS (none, low, moderate, and high). For the analyses, we combined those who reported being HIV-positive and those with high risk. Ownership of radio and television was measure by a set of dichotomous variables.

We also included dummy variables indicating whether the respondent desires a child within the next two years, and whether his or her last partner was a casual partner.

### Statistical methods

As most of the variables were categorical χ^2^-tests were used to compare their distributions for the female and male samples. For the few parametric variables, independent sample *t*-tests were performed to compare female and male samples.

When examining the effect of program exposure on condom use, it is possible that the same unobserved exogenous factors that affect condom use also affect program exposure, as well as that condom users may seek out these programs. For instance, someone who intends to use condoms to avoid HIV infection may actively seek out sources of information about condom use and where to obtain them. Standard single stage regression techniques always assume that all predictor variables are exogenous to the model. When program exposure, however, is endogenous and the error terms of program exposure and condom use variables are correlated, the estimate for the effect of program exposure on condom use may be biased. Researchers can avoid this pitfall by using two-stage regression models in those instances when program exposure shows substantial endogeneity [35-37].

The proposed two-stage model first requires estimating program exposure using a set of exogenous variables. As the program exposure indicators used here are count variables, we used Poisson regression for this first step. In the second step, the estimated values for program exposure are used in the model for reproductive health behaviors rather than the observed program exposure. As our condom use measures are dichotomous, logistic regression is used for this second step. To test for endogeneity Bollen et al. [35] suggested a simple test consisting of including the residual for program exposure of the stage one equation together with the observed program exposure variable in the stage two equation. The *t*-test for the effect of the residual is used to test for endogeneity. Where no significant endogeneity in program exposure was observed, the standard one-stage model is used.

To estimate a two-step model, one needs to assure that the model is identified. In a strict sense this condition is fulfilled because the sets of exogenous variables in the two structural equations are not identical. That is, the exogenous predictors of program exposure and of condom use are not all the same. However, ideally one would also like that the variable excluded from the structural model for condom use are unrelated to these outcome variables. To test the identification of the model we used a test proposed by Bollen et al. [35], based on the comparison of the goodness-of-fit of the unrestricted reduced models and the structural models for condom use in which the predicted program exposure was substituted for observed exposure. This test indicated that there were no identification problems for the either the male or female sample.

To estimate program exposure, the following variables were included in the model: age, residence, education, number of partners, perceived AIDS risk, and the media exposure indicators. In the model for condom use, the following variables were included: program exposure, age, religion, residence, education, number of partners, perceived AIDS risk, and the proximate determinants of condom use. Because contraceptive use could potentially confound the relationship between program exposure and condom use, we also tested a model that included contraceptive use. Because the effect of contraceptive use was not significant, and because data on contraceptive use were collected for the female sample only, this variable was not included in the final model.

The results are presented in the form of odds ratios. In the case of parametric variables, such as the number of reproductive health programs respondents were exposed to, we also discuss the interdecile odds ratio (OR_D_). The interdecile odds ratio rank-orders respondents in terms of level of exposure, and then estimates likelihood that the outcome was experienced by those 10% of respondents who had the highest level of exposure, relative to the likelihood that it was experienced by the 10% of the respondents with the lowest levels of exposure.

### Sample description

Table 1 shows the sample characteristics. The female and male samples are significantly different on most socio-demographic characteristics, although many of the differences are small. Over two thirds of respondents lived in rural areas, while 18% lived in a town, 6% in a small city and 9% in the capital Lusaka. The male sample was on the average 2.5 years older than the female one due to the different eligibility criteria for the two samples. Women are more likely than men to have had no formal education (14% vs. 5%) and less likely than men to have had secondary or higher education (26% vs. 42%). Ownership of radio and TV was also significantly higher among men than among women. While 50% of men reported owning a radio and 22% a TV, among women only 43% and 18% reported owning one, respectively.

**Table 1:** Descriptive statistics (unweighted samples)

	Women	Men	P
Age			
15–19	15.4%	15.4%	0.000
20–24	23.1%	16.6%	
25–29	19.7%	18.0%	
30–34	14.2%	14.2%	
35–39	11.5%	12.6%	
40–44	8.9%	8.8%	
45–49	7.2%	5.8%	
50–54		4.9%	
55–59		3.8%	
			
De facto place of residence			
Lusaka	9.4%	8.9%	0.333
Other Urban	22.7%	24.2%	
Rural	67.9%	67.0%	
			
Religion			
Catholic	22.4%	23.9%	0.000
Protestant	75.3%	72.0%	
Other	2.4%	4.1%	
			
Highest educational level			
No education	14.0%	5.4%	0.000
Primary	60.1%	52.7%	
Secondary and Higher	25.9%	41.8%	
			
Perceived risk of HIV/AIDS			
No risk at all	28.8%	37.2%	0.000
Small	17.1%	19.4%	
Moderate	26.9%	17.7%	
Great or Has AIDS	27.2%	25.8%	
			
Number of partners in the past 12 months (including husband)	0.87	1.24	0.000
			
Owns Radio			
No	57.1%	50.4%	0.000
Yes	42.9%	49.6%	
			
Owns TV			
No	81.9%	78.1%	0.000
Yes	18.1%	21.9%	
			
Desires child within 2 years			
No	82.2%	69.9%	0.000
Yes	17.8%	30.1%	
			
Last partner was casual partner			
No	98.9%	89.1%	0.000
Yes	1.1%	10.9%	
		
N of Cases	6,782	1,928	

Men report higher levels of risky sexual behavior than females. Where only 2% of the women reported more than one sex partner in the past year, 20% of men did. Similarly, 11% of men but only 1% of women report that their last sexual partner was a casual partner. Nevertheless, men were more likely than women to consider themselves at no or at low risk of HIV/AIDS. Forty-six percent of females and 57% of males considered themselves at no or low risk of getting AIDS. A significantly higher percentage of men than women desired a child within the next two years (30% vs. 18%).

**Figure 1:**
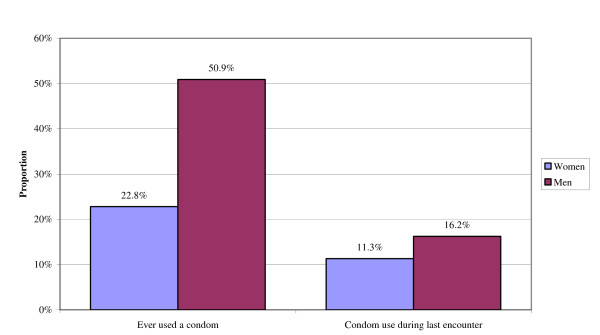
Condom Use, by Gender of respondent (Sexually Active Respondents Only).

Women reported significantly less condom use than men (Figure 1). Only 23% of the sexually active women reported ever having used a condom, compared to 51% of men. Similarly, 11% of women and 16% of men report condom use during last intercourse.

## Results

### Levels of program exposure

**Figure 2:**
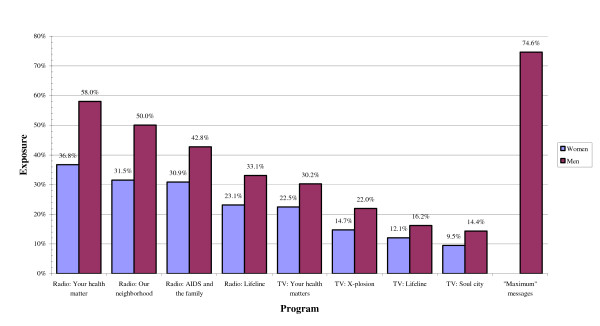
Percentage who recall exposure to reproductive health radio and television programs in the past six months, and recall of *Maximum *condom advertisements.

Figure 2 shows the percentage of females and males who reported recalling exposure to any of the four radio reproductive health programs, as well as exposure to each of the four television programs on reproductive health. For males, we also show the percentage who recalled messages about the social marketed *Maximum *condoms.

The radio program associated with the highest levels of recall was *Your Health Matters*, followed by *Our Neighborhood *and *AIDS and the Family*. Figure 1 shows that 58% of males and 36% of females recalled hearing *Your Health Matters *in the past six months. The television program *Your Health Matters *also triggered the highest recall (30% of men and 23% of women), followed by *X-plosion*. For all programs, women reported lower exposure than men. On average, women reported having seen or heard 1.8 out of the 8 programs listed, while men had seen or heard 2.7 of these programs (not shown). Three quarters of the male respondents (75%) reported having been exposed to messages for the socially marketed *Maximum *condoms.

Table 2 presents the results of Poisson regression analyses on our program exposure variables. The purpose of these analyses is twofold: 1) to identify determinants of program exposure, and 2) in the first stage of two-stage regression to generate both the instrumental variable and the residual variable required for the endogeneity test. The included socio-demographic factors affect almost all the program exposure indicators, but the effects vary substantially. For instance, for both men and women, age had a significant positive effect on the total number of programs exposed to and on the number of radio programs exposed to. However, age seems to have a small negative effect on the number of TV programs exposed to, as well as on exposure to information about *Maximum *condoms.

**Table 2:** Poisson regression results for program exposure indicators, by gender

	Females			Males			
		
	Total	Radio programs	TV programs	Total (radio and TV)	Radio programs	TV programs	"Maximum" sources
Age	0.002*	0.006***	-0.005**	0.005***	0.008***	-0.004	-0.008***
Residence (ref: rural)							
Capital	0.881***	0.593***	1.736***	0.510***	0.212***	1.242***	0.377***
Other urban	0.637***	0.457***	1.364***	0.346***	0.130**	0.987***	0.129*
Education (ref: no formal education)							
Primary	0.835***	0.812***	0.982***	0.578***	0.570***	0.735**	0.465***
Secondary and higher	1.329***	1.224***	1.666***	0.980***	0.863***	1.459***	0.807***
Number of partners in past 12 months	0.018	0.030	-0.008	0.019	0.019	0.029	0.031*
Perceived AIDS risk (ref: None)							
Small	-0.038	-0.070*	0.019	0.033	-0.026	0.174*	0.078
Moderate	-0.071**	-0.094**	-0.020	-0.108*	-0.090	-0.155	-0.005
High + Has AIDS	0.174***	0.207***	0.100*	0.094**	0.118**	0.031	0.034
Own Radio	0.481***	0.650***	-0.009	0.387***	0.473***	0.145*	0.160***
Owns TV	0.449***	0.103**	1.225***	0.278***	-0.007	0.829***	0.181**
Constant	-1.254***	-1.504***	-3.143***	-0.416***	-0.666***	-2.249***	-0.328*

Pseudo-R2	26.6%***	15.7%***	40.9%***	16.7%***	7.4%***	31.4%***	6.2%***
N	6,782	6,782	6,782	1,928	1,928	1,928	1,928

Significance: *: *p *< 0.050; **: *p *< 0.010; ***: *p *< 0.001

In general, urban respondents were likely to have been exposed to a larger number of reproductive health programs than rural respondents. However, the effect of urbanization on program exposure is substantially larger for exposure to TV programs than for radio programs. The effect of urbanization was also higher among women than among men. Exposure to these reproductive health programs also increased with the education level of the respondents; again the effects were larger among women than among men.

The indicators of risk behavior had little impact on program exposure. For example, the reported number of partners in the past 12 months only affects exposure to the *Maximum *campaign. Perceived AIDS risk also had only little effect on program exposure. Among women, only those who considered themselves to be at high risk for AIDS or who reported having AIDS had a significantly higher level of program exposure, while those who reported a moderate risk tended to have lower program exposure. Among men these effects were substantially smaller, and often not significant.

As anticipated, program exposure was affected by the respondents' ownership of rado and TV. Women who owned a radio tended to have a higher total program exposure and exposure to radio programs than women who did not own a radio. Radio ownership among women, however, did not affect exposure to TV programs. Among men, radio ownership had a positive effect on all program exposure indicators, but the effects were substantially larger for total exposure and exposure to radio programs than for exposure to TV programs and exposure to *Maximum *sources. TV ownership among women had a significant positive effect on all program exposure variables, but again the effect was substantially larger for exposure to TV programs than for the other variables. Similar effects were observed for men, except that the effect of exposure to radio programs was not significant.

### Effect of campaign exposure on condom use

Because the possibility exists that the observed associations between program exposure and condom use are affected by the endogeneity of program exposure, we first tested for the presence of endogeneity [see [35]]. The results of these analyses indicate that no endogeneity problems were identified, and that a one-stage model can be used. For ever condom use the significance levels of the residual terms varied from 0.369 to 0.953 for men, and from 0.405 to 0.945 for women. For condom use at last intercourse the significance levels of the residuals varied from 0.043 to 0.921 for men and from 0.136 to 0.983 for women. Only for a single model did the significance level fall below 0.100. Overall, therefore, one can conclude that endogeneity is not a problem in these equations. In the tables only the one-stage logistic regression results are shown. Tables including the two-stage results are available from the authors upon request.

### Effect on ever use of condoms

Our estimates of the effect of exposure to social marketing and health communication programs on the likelihood that women ever used condoms are shown in Table 3. The results show that exposure to radio and television programs about family planning and HIV/AIDS had a significant but small positive effect on the likelihood that women have tried condoms (OR = 1.06).

**Table 3:** Estimates of the impact of program exposure on the odds of ever having used a condom, female sample

OR (95% CI)	Total exposure	Radio programs	TV programs
Exposure			
Total	1.059*** (1.029 – 1.090)		
Radio programs		1.079*** (1.036 – 1.124)	
TV programs			1.082** (1.021 – 1.147)
Age	0.964*** (0.957 – 0.971)	0.964*** (0.957 – 0.971)	0.965*** (0.958 – 0.972)
Religion (ref: Protestant)	**	**	**
Catholic	0.831* (0.716 – 0.965)	0.833* (0.717 – 0.966)	0.832* (0.717 – 0.966)
Other	0.543* (0.318 – 0.928)	0.544* (0.318 – 0.930)	0.537* (0.314 – 0.917)
Residence (ref: rural)	***	***	***
Capital	1.699*** (1.382 – 2.087)	1.829*** (1.505 – 2.223)	1.741*** (1.407 – 2.154)
Other urban	1.261** (1.079 – 1.475)	1.313*** (1.130 – 1.527)	1.298** (1.109 – 1.519)
Education (ref: No formal education)	***	***	***
Primary	2.090*** (1.632 – 2.675)	2.079*** (1.623 – 2.663)	2.150*** (1.680 – 2.751)
Secondary & higher	4.798*** (3.669 – 6.274)	4.903*** (3.756 – 6.399)	5.100*** (3.913 – 6.647)
Number of partners past 12 months	2.260*** (1.917 – 2.665)	2.253*** (1.911 – 2.657)	2.261*** (1.918 – 2.666)
Perceived AIDS risk (ref: None)			
Small	1.203* (1.002 – 1.444)	1.206* (1.005 – 1.447)	1.200 (1.000 – 1.440)
Moderate	1.183* (1.006 – 1.392)	1.185* (1.007 – 1.394)	1.178* (1.001 – 1.385)
High + has AIDS	1.008 (0.855 – 1.190)	1.006 (0.853 – 1.187)	1.022 (0.867 – 1.206)
Wants child in next 2 years	0.703*** (0.595 – 0.830)	0.702*** (0.594 – 0.829)	0.706*** (0.598 – 0.834)
Last partner was casual	0.646 (0.360 – 1.160)	0.647 (0.361 – 1.161)	0.636 (0.355 – 1.142)
Constant	0.127*** (0.089 – 0.183)	0.127*** (0.089 – 0.182)	0.127*** (0.089 – 0.182)

OR_D_	1.41	1.36	1.27

Pseudo-R^2^	10.0%***	10.0%***	9.9%***

Significance: *: *p *< 0.050; **: *p *< 0.010; ***: *p *< 0.001

Among women the OR_D_-s of total exposure, radio program exposure and TV program exposure are 1.41, 1.36 and1.27, respectively, indicating that those with the highest levels of exposure are roughly 30% more likely than those with the lowest levels of exposure to have tried condoms.

Older women are less likely than younger ones and Protestant respondents more likely than Catholics or members of other religions to have ever used condoms. The likelihood of ever having used condoms increases with the level of urbanization and education. The likelihood that a woman has tried condoms is also significantly higher for those who reported a larger number of sexual partners. Women who reported wanting to have a child in the next two years are less likely than other women to have ever used a condom.

Table 4 shows the results of similar analyses for men. The results show that exposure to radio and television programs about family planning and HIV/AIDS had a positive effect on the likelihood that males had ever used condoms (OR = 1.16). Exposure to *Maximum *messages had a very strong positive effect on male respondents' ever use of condoms (OR = 1.48).

**Table 4:** Estimates of the impact of program exposure on the odds of ever having used a condom, male sample

OR (95% CI)	Total	Radio	TV	"Maximum" sources
Exposure				
Total	1.159*** (1.104 – 1.216)			
Radio programs		1.245*** (1.164 – 1.331)		
TV programs			1.141** (1.040 – 1.251)	
"Maximum" sources				1.475*** (1.329 – 1.637)
Age	0.963*** (0.954 – 0.973)	0.962*** (0.952 – 0.971)	0.966*** (0.957 – 0.975)	0.969*** (0.959 – 0.978)
Religion (ref: Protestant)	*	*	*	*
Catholic	0.764* (0.604 – 0.967)	0.755* (0.596 – 0.955)	0.788* (0.624 – 0.995)	0.769* (0.607 – 0.974)
Other	0.606 (0.353 – 1.040)	0.600 (0.350 – 1.030)	0.608 (0.357 – 1.037)	0.613 (0.358 – 1.051)
Residence (ref: rural)	**	***	***	***
Capital	1.775** (1.200 – 2.626)	2.171*** (1.485 – 3.174)	1.921** (1.284 – 2.876)	1.782** (1.209 – 2.627)
Other urban	1.365* (1.051 – 1.774)	1.580*** (1.228 – 2.032)	1.486** (1.132 – 1.949)	1.525** (1.183 – 1.966)
Education (ref: No formal education)	***	***	***	***
Primary	1.644* (1.015 – 2.662)	1.605 (0.988 – 2.608)	1.787* (1.109 – 2.878)	1.606 (0.989 – 2.607)
Secondary & higher	3.030*** (1.829 – 5.019)	3.125*** (1.886 – 5.177)	3.655*** (2.222 – 6.010)	2.952*** (1.781 – 4.892)
Number of partners past 12 months	2.412*** (2.029 – 2.867)	2.407*** (2.026 – 2.861)	2.402*** (2.023 – 2.852)	2.387*** (2.007 – 2.837)
Perceived AIDS risk (ref: None)				
Small	1.219 (0.924 – 1.607)	1.259 (0.955 – 1.661)	1.211 (0.920 – 1.593)	1.185 (0.897 – 1.566)
Moderate	1.224 (0.910 – 1.648)	1.228 (0.912 – 1.654)	1.197 (0.891 – 1.609)	1.193 (0.884 – 1.611)
High + has AIDS	1.054 (0.815 – 1.363)	1.052 (0.813 – 1.360)	1.087 (0.843 – 1.403)	1.084 (0.838 – 1.402)
Wants child in next 2 years	1.365** (1.092 – 1.706)	1.331* (1.064 – 1.663)	1.345** (1.078 – 1.680)	1.337* (1.069 – 1.672)
Last partner was casual	1.191 (0.846 – 1.676)	1.196 (0.850 – 1.685)	1.132 (0.807 – 1.588)	1.165 (0.826 – 1.643)
Constant	0.306*** (0.168 – 0.558)	0.304*** (0.166 – 0.554)	0.325*** (0.180 – 0.589)	0.229*** (0.124 – 0.422)

OR_D_	2.42	2.40	1.49	3.21

Pseudo-R^2^	14.7%***	14.9%***	13.6%***	15.4%***

Significance: *: *p *< 0.050; **: *p *< 0.010; ***: *p *< 0.001

The interdecile odds ratios for radio and television dramas show that men with the highest levels of exposure are 2.4 and 1.5 times more likely, respectively, than those with the lowest levels of exposure to have ever used a condom. Males with the highest levels of exposure to messages about *Maximum *brand condoms are 3.2 times more likely than those with the lowest levels to have tried condoms (OR_D _= 3.21).

The likelihood that males have ever used condoms decreases with age, and is lower for males who are not Protestants. The likelihood increases with level of urbanization, education, and the number of sexual partners. Contrary to women, ever use of condoms is higher among males who wish to have a child in the next two years. Perceived AIDS risk, and type of last sexual partner, did not have an effect on males' ever use of condoms.

### Effect on condom use in last intercourse

Table 5 shows that exposure to radio or television programs about family planning and HIV/AIDS had no significant impact on the likelihood that women used a condom in last intercourse.

**Table 5:** Estimates of the impact of program exposure on the odds of condom use during last intercourse, female sample

OR (95% CI)	Total exposure	Radio programs	TV programs
Exposure			
Total	1.006 (0.968 – 1.046)		
Radio programs		1.004 (0.950 – 1.061)	
TV programs			1.017 (0.940 – 1.100)
Age	0.964*** (0.955 – 0.973)	0.964*** (0.955 – 0.973)	0.964*** (0.955 – 0.973)
Religion (ref: Protestant)			
Catholic	0.890 (0.733 – 1.081)	0.890 (0.733 – 1.081)	0.890 (0.733 – 1.081)
Other	0.656 (0.338 – 1.272)	0.656 (0.338 – 1.272)	0.655 (0.338 – 1.271)
Residence (ref: rural)	*	*	*
Capital	1.341* (1.026 – 1.753)	1.357* (1.053 – 1.750)	1.326* (1.004 – 1.752)
Other urban	0.942 (0.762 – 1.165)	0.949 (0.773 – 1.166)	0.937 (0.756 – 1.161)
Education (ref: No formal education)	***	***	***
Primary	1.223 (0.928 – 1.612)	1.225 (0.929 – 1.615)	1.226 (0.931 – 1.614)
Secondary & higher	2.490*** (1.830 – 3.387)	2.509*** (1.848 – 3.406)	2.489*** (1.839 – 3.369)
Number of partners past 12 months	5.111*** (3.996 – 6.538)	5.109*** (3.994 – 6.535)	5.112*** (3.996 – 6.539)
Perceived AIDS risk (ref: None)	***	***	***
Small	0.920 (0.739 – 1.145)	0.920 (0.739 – 1.145)	0.919 (0.739 – 1.144)
Moderate	0.617*** (0.501 – 0.759)	0.617*** (0.501 – 0.759)	0.616*** (0.501 – 0.759)
High + has AIDS	0.495*** (0.398 – 0.617)	0.496*** (0.398 – 0.617)	0.496*** (0.398 – 0.617)
Wants child in next 2 years	0.529*** (0.419 – 0.668)	0.529*** (0.419 – 0.668)	0.529*** (0.419 – 0.668)
Last partner was casual	1.095 (0.595 – 2.015)	1.094 (0.594 – 2.013)	1.094 (0.594 – 2.012)
Constant	0.075*** (0.048 – 0.119)	0.075*** (0.048 – 0.119)	0.076*** (0.048 – 0.119)

OR_D_	1.04	1.02	1.05

Pseudo-R^2^	9.3%***	9.3%***	9.3%***

Significance: *: *p *< 0.050; **: *p *< 0.010; ***: *p *< 0.001

Again older women were less likely to have used a condom during their last intercourse than younger women. Religion had no discernable effect on the likelihood of having used a condom in last intercourse. Women living in the capital were slightly more likely than rural women to have used a condom at their last sexual intercourse. Women's level of education and number of sexual partners increased the likelihood that they used a condom in last intercourse, while perceived HIV/AIDS risk and the desire to have a child in the next two years decreased this likelihood.

Table 6 shows the effect of exposure to social marketing and health communication programs on the likelihood that males used a condom in last intercourse. Unlike the case for females, the results show that exposure to such programs had a significant positive effect on condom use in last intercourse (OR = 1.10). Exposure to radio programs on family planning and HIV/AIDS had a significant positive effect (OR = 1.16), but exposure to television programs on such topics had no significant effect. Exposure to messages for the socially marketed *Maximum *brand condoms also increased the likelihood of condom use in last intercourse (OR = 1.23).

**Table 6:** Estimates of the impact of program exposure on the odds of condom use during last intercourse, male sample

OR (95% CI)	Total exposure	Radio programs	TV programs	"Maximum" sources
Exposure				
Total	1.095** (1.030 – 1.164)			
Radio programs		1.161** (1.060 – 1.272)		
TV programs			1.079 (0.964 – 1.207)	
"Maximum" sources				1.232** (1.084 – 1.401)
Age	0.945*** (0.931 – 0.959)	0.943*** (0.929 – 0.958)	0.947*** (0.933 – 0.961)	0.947*** (0.934 – 0.962)
Religion (ref: Protestant)				
Catholic	1.111 (0.823 – 1.501)	1.111 (0.823 – 1.500)	1.123 (0.833 – 1.516)	1.115 (0.825 – 1.505)
Other	0.858 (0.402 – 1.832)	0.859 (0.403 – 1.831)	0.862 (0.405 – 1.833)	0.870 (0.408 – 1.855)
Residence (ref: rural)		*		
Capital	1.432 (0.922 – 2.224)	1.634* (1.074 – 2.486)	1.535 (0.967 – 2.436)	1.492 (0.971 – 2.293)
Other urban	1.220 (0.878 – 1.695)	1.334 (0.976 – 1.824)	1.297 (0.922 – 1.826)	1.335 (0.977 – 1.824)
Education (ref: No formal education)	*	**	**	**
Primary	1.213 (0.595 – 2.474)	1.185 (0.580 – 2.420)	1.292 (0.635 – 2.627)	1.201 (0.589 – 2.448)
Secondary & higher	1.842 (0.885 – 3.833)	1.859 (0.896 – 3.854)	2.109* (1.020 – 4.361)	1.851 (0.893 – 3.835)
Number of partners past 12 months	1.195** (1.067 – 1.338)	1.192** (1.064 – 1.337)	1.199** (1.073 – 1.341)	1.195** (1.068 – 1.337)
Perceived AIDS risk (ref: None)				
Small	1.383 (0.968 – 1.976)	1.398 (0.979 – 1.998)	1.388 (0.972 – 1.982)	1.359 (0.951 – 1.942)
Moderate	1.347 (0.923 – 1.965)	1.340 (0.918 – 1.955)	1.333 (0.915 – 1.943)	1.300 (0.891 – 1.897)
High + has AIDS	1.061 (0.752 – 1.496)	1.049 (0.743 – 1.479)	1.086 (0.771 – 1.530)	1.076 (0.763 – 1.516)
Wants child in next 2 years	0.693* (0.503 – 0.954)	0.683* (0.496 – 0.940)	0.685* (0.497 – 0.942)	0.677* (0.492 – 0.932)
Last partner was casual	2.670*** (1.879 – 3.795)	2.700*** (1.898 – 3.841)	2.592*** (1.828 – 3.675)	2.610*** (1.837 – 3.710)
Constant	0.305** (0.131 – 0.708)	0.302** (0.130 – 0.703)	0.308** (0.133 – 0.713)	0.266** (0.114 – 0.620)

OR_D_	1.72	1.82	1.26	1.87

Pseudo-R^2^	11.9%***	12.1%***	11.5%***	12.0%***

Significance: *: *p *< 0.050; **: *p *< 0.010; ***: *p *< 0.001

In the male sample the OR_D_-s are slightly higher with values of 1.71, 1.82, 1.26, and 1.87, respectively for total program exposure, exposure to radio programs, exposure to TV programs, and exposure to *Maximum *messages. In other words, men with the highest levels of exposure are 26–87% more likely than those with the lowest levels of exposure to have used a condom in last intercourse.

For men, the likelihood of having used a condom in last intercourse decreases slightly with age and with the desire to have a child in the near future. The likelihood is higher for males who had a larger number of sexual partners, and whose last partner was a casual partner.

## Discussion

The purpose of this paper was to assess the reach of selected social marketing and reproductive health communication activities in Zambia, and to assess their impact on condom use. Specifically, we examined the reach and impact of several radio and television programs on family planning and HIV prevention, as well as the reach of communication for the social marketed *Maximum *brand condoms.

Our study is subject to several limitations. As in most studies, the measures of campaign recall and behavioral outcomes in this study are based on self-reported information, and therefore subject to recall errors and reporting biases. We also acknowledge that the goodness of fit of our models is low. Also, because our data are restricted to the main health communication campaigns, we are unable to assess the reach and impact of the total spectrum of health communication campaigns in Zambia.

Despite these limitations, our study yield several important findings. Exposure to the reproductive health-related radio and television programs examined appears to have been very high. The most popular radio program was *Your Health Matters*, which produced 58% recall among males and 36% among females. Recall was also high for the radio programs *Our Neighborhood *and *AIDS and the Family*, which were heard by over one third of respondents. The televised version of *Your Health Matters *also had high recall (30% among men; 23% among women), as did *X-plosion*. On average, women reported recalling 1.8 of the eight programs studies, while men recalled 2.7 programs. The large majority of male respondents (75%) recalled exposure to messages about the socially marketed *Maximum *condoms.

The data suggest that exposure to these health communication campaigns had a significant effect on the likelihood that females and males ever used condoms, although the effect was fairly small among women. Males with the highest levels of exposure to reproductive health-related radio programs and to *Maximum *messages were more than twice as likely as those with low levels of exposure to have ever used condoms. The effect of campaign exposure on condom use in last intercourse varied by gender. Exposure to these programs had no effect on the likelihood that women used a condom in last intercourse, but the data suggest that it significantly increased the likelihood that men did. Men with the highest levels of exposure were roughly 50% more likely than those with the lowest levels of exposure to have used a condom in last intercourse. Differentiating between the types of campaigns shows that exposure to radio programs and *Maximum *condom messages appears to have had significant effects, while exposure to television programs did not.

## Conclusion

In conclusion, the evidence shows that social marketing condom advertisements and radio and television programs on family planning and HIV/AIDS have succeeded in reaching a large fraction of the Zambian population. The results further suggest that exposure to these campaigns has a strong effect on condom use, even after controlling for other factors. However, the data also indicate that radio programs and *Maximum *condom advertisements may be more effective than television programs. The data also show that the effect of these campaigns on condom use is considerably stronger for males than females. These findings suggest that future reproductive health communication campaigns should consider investing in radio programming, as such programs may be more effective than those investing in television programming. The findings also suggest that future social marketing and reproductive health communication campaigns should seek to increase their impact among women, perhaps by focusing on specific constraints that prevent females from using condoms.

The current study allows us to estimate the overall effect of program exposure on condom use. However, it does not tell us which aspects of the program content or which specific messages actually affect condom use, nor does it clarify the processes through which program exposure affects condom use.

Future research should assess which specific message content of radio and television campaigns has the highest recall, and investigate which specific messages – or combinations of messages – have the largest effect on condom use. Doing so will require having detailed information about program content and about broadcast schedules, which will facilitate the design of survey questionnaires that better capture the frequency and nature of program exposure. This in turn will require close collaboration between program staff and researchers. Evaluations of the impact of mass media campaigns can be further strengthened by using a panel study design, including both a pre- and a post-intervention assessment. Although it is typically not feasible to implement such studies on a large scale, it may be helpful to implement them on a smaller scale for the specific purpose of enhancing our understanding of what message contact works best.

## Competing interests

The author(s) declare that they have no competing interests.

## Authors' contributions

DM conceived of the study and drafted the background section and discussion and conclusions. RV developed the study design, carried the statistical analysis, and drafted the methods and results sections. Both authors read and approved the final manuscript.

## Pre-publication history

The pre-publication history for this paper can be accessed here:


